# Active immunization with Tocilizumab mimotopes induces specific immune responses

**DOI:** 10.1186/s12896-015-0161-9

**Published:** 2015-06-02

**Authors:** Lin Yang, Rui Xing, Changhong Li, Yuan Liu, Lin Sun, Xiangyuan Liu, Yongfu Wang

**Affiliations:** Department of Rheumatology and Immunology, Peking University Third Hospital, No.49, HuaYuan (North) Road, Beijing, 100191 People’s Republic of China; Department of Rheumatology, the First Affiliated Hospital of Baotou Medical College, No.41, LinYin Road, Baotou, 014010 People’s Republic of China

**Keywords:** Rheumatoid arthritis, Tocilizumab, Mimotope, IL-6, Phage display

## Abstract

**Background:**

Tocilizumab is a humanized monoclonal antibody showing high-affinity binding to both soluble interleukin-6 receptor (sIL-6R) and membrane bound IL-6R (mIL-6R), thereby preventing pro-inflammatory effects of IL-6. However, therapeutic antibodies still have practical limitations. To overcome these limitations, we generated Tocilizumab specific epitope mimics by using the phage display technology and tested whether the peptide mimics could induce similar humoral responses in mice immunized with the peptides.

**Results:**

Seven phage mimics were obtained by using phage display peptide library. Four phage mimics (YHTTDKLFYMMR, YSAYEFEYILSS, KTMSAEEFDNWL and LTSHTYRSQADT) were shown to mimic Tocilizumab epitope using immunoassays. The mimotopes were conjugated to immunogenic carrier proteins and used to intraperitoneally immunize BALB/c mice. Sera from the mimotopes immunized mice not only showed specific binding to recombinant IL-6R, but can also IL-6R expressed in Hela, U-937, Jurkat cell lines and in fibroblast-like synoviocytes from patients with RA (FLS-RA). Furthermore, sera from mice immunized with mimotopes-KLH conjugate could reduce the level of phosphorylated- signal transducers and activator of transcription (STAT3), STAT3, phosphorylated- extracellular signal-regulated kinase (Erk) 1/2 and Erk1/2 in HeLa and Jurkat cells. Antibody-dependent cellular cytotoxicity (ADCC) assay showed that antibodies induced by mimotopes-KLH conjugate could elicit specific lysis in Hela and U-937 cells.

**Conclusions:**

From phage display library, we successfully isolated four Tocilizumab mimotopes which induced specific humoral and cellular reponses *in vitro* and *in vivo*.

## Background

Rheumatoid arthritis (RA) is an autoimmune inflammatory disease characterized by abnormal immune responses which lead to persistent synovitis and ultimately progressive destruction of cartilage and bone in multiple joints. Among great progress in underlying the pathogenesis of RA, overexpression of inflammatory cytokines recruited by activated synovial fibroblasts is thought to play an important role in synovial hyperplasia and joint destruction [1]. Interleukin-6 (IL-6), a pleiotropic cytokine, contributes to the regulation of the immune response, inflammation, and hematopoiesis [2, 3]. Disruption of IL-6 regulation has been demonstrated to play a crucial role in pathogenesis of rheumatoid arthritis [3, 4], including promoting leukocyte recruitment to the site of inflammation [5], helping naive T cell differentiation to T helper 17 cells [6], stimulating osteoclast maturation and activation via receptor activator of nuclear factor-Κb ligand (RANKL) [7], promoting angiogenesis by increasing production of vascular endothelial growth factor (VEGF) [8]. These findings suggest that targeting IL-6 can be used as a therapeutic strategy and is a promising alternative in the treatment of RA.

Tocilizumab is a humanized monoclonal antibody (mAb) that specifically targets both soluble interleukin-6 receptor (sIL-6R) and membrane bound interleukin-6 receptor (mIL-6R) with a high affinity, thereby preventing pro-inflammatory effects of IL-6 [9]. To present, the application of Tocilizumab is licensed for the treatment of RA [10, 11], systemic juvenile idiopathic arthritis (sJIA) [12, 13] and Castleman’s disease [14, 15] and represents a favorable efficacy in these diseases. However, therapeutic antibodies still have practical limitations. For instance, obstacles in production, high dose in application lead to a relatively high price and persist blocking activity needs repeatedly administration.

Active immunization using epitope peptides to initiate the ongoing production of the desired antibody type would be an attractive alternative. However, due to immune tolerance, the use of a natural epitope peptide alone is inefficient in inducing efficient immune response [16]. Mimotopes are small peptides that structurally mimic a given antibody-binding site but are composed of different amino acids [17, 18]. Active immunization using mimotopes would induce antibodies to recognize the mimicked epitope. Notably, mimotopes can be isolated from phage display peptide libraries [19]. In current study, to make a substitution of Tocilizumab, we screened and identified four peptides that can mimic the natural epitope of Tocilizumab by using the phage display technology.

## Methods

### Cell lines and anti-IL-6R antibody

Human epithelial adenocarcinoma cell line HeLa was kindly provided by Dr. Han (Center for Human Disease Genomics, Department of Immunology, College of Basic Medical Sciences, Peking University). Human histiocytic lymphoma cell line U-937 and human acute T-cell leukemia cell line Jurkat were provided by medical research center, Peking University Third Hospital. Hela, U-937 and Jurkat cells were grown in RPMI 1640 medium (ATCC, Manassas, VA) supplemented with 10 % fetal bovine serum (FBS, HyClone Laboratories, Logan, UT), 100U/ml penicillin and 100 mg/ml streptomycin. Fibroblast-like synoviocytes from patients with RA (FLS-RA) were isolated from three RA patients at the time of joint replacement surgery. Patients were informed consent and the approval was obtained from the Ethics Committee of Baotou Medical College. FLS-RA cells were maintained in 10 % FBS, 2 mM L-glutamine, 3 mM NaHCO_3_, 100U/ml penicillin and 100 mg/ml streptomycin. FLS-RA cells were used to test from passage 3 to 8. For signaling pathway detection, cells were grown at 37°C with medium containing 10 % FBS. Two days later, the medium was replaced by FBS-free medium with sera from mimotopes immunized mice with a dilution 1:100 or Tocilizumab (10 μg/ml, Roche Co. Ltd, Shanghai, China) at 37°C for 2 h. Then the cells were incubated with human IL-6 (40 ng/ml, R&D Systems, Minneapolis, MN) for 15 min at 37°C. Total cell lysates were prepared and the protein concentration was determined (BCA Protein Assay Kit; Pierce, Rockford, IL). The lysates were aliquoted and stored at −80°C.

### Biopanning of PH.D-12 phage display library

The Ph.D-12 library was purchased from New England Bio-Labs Inc. (1.5 × 10^13^ pfu/ml, complexity = 2.7 × 10^9^ transformants; Beverly, MA, USA). Four rounds of biopanning procedures were performed according to the manufacturer’s instructions. Briefly, a 96-well plate (MaxiSorp; Thermo Fisher Scientific, Shanghai, China) was coated with 100 μl Tocilizumab [100 μg/ml in 0.1 M NaHCO_3_ (pH 8.6)] overnight at 4°C. Wells were then washed 10 times with TBST buffer (50 mM Tris, 150 mM NaCl, pH 7.5 containing 0.1 % Tween-20), filled with 300 μl of blocking buffer [0.1 M NaHCO_3_ pH 8.6, 5 mg/ml bovine serum albumin (BSA)] and incubated for 2 h at 37°C. In the first round of biopanning, 10 μl of the phage (1.5 × 10^11^ pfu) isolated from the initial library in 100 μl of TBST was incubated with the coated Tocilizumab for 1 h at 37°C. After repeated washing with TBST, the bound phage was eluted with 100 μl of 0.2 M Glycine–HCl (pH 2.2) and neutralized with 15 μl of 1 M Tris–HCl (pH 9.1). The eluate was used for amplification and titration in an *Escherichia coli* ER2738 culture. The recovered phage was subjected to three additional rounds of biopanning with Tocilizumab and isotype control (purified human IgG, R&D Systems, Minneapolis, MN). The eluate from the fourth round of screening was titrated, and blue clones were randomly selected and amplified by infecting ER2738.

### DNA sequencing

Single-stranded phage DNA was prepared according to the Ph.D-12 phage display library manufacturer’s instructions and was subsequently sequenced by Invitrogen Inc. (Shanghai, China).

### Specificity enzyme-linked immunosorbent assay (ELISA)

Ninety-six-well plates (MaxiSorp; Thermo Fisher Scientific, Shanghai, China) were coated with Tocilizumab, an isotype-matched control antibody [100 μg/ml in 0.1 M NaHCO_3_ (pH 8.6)] and 2 % BSA overnight at 4°C. The plates were then washed with TBS containing 0.5 % Tween-20 and subsequently blocked using TBS containing 5 % dry milk. 1 × 10^9^ pfu of amplified phages after the fourth round in TBS containing 5 % dry milk were incubated with the coated antibodies. The bound phage particles were detected with a peroxidase-conjugated mouse anti-phage M13 monoclonal antibody (Pharmacia, Peapack, USA). The reaction was then developed with 2, 2-azino-bis-(3-ethylbenzthiazoline-6-sulphonic acid) (ABTS, SIGMA, St. Louis, MO) as a substrate. The optical density at 405 nm was measured using an ELISA reader (Thermo Fisher Scientific, Shanghai, China). The specificity ELISA was performed in triplicate.

The same procedure was also used to examine the binding specificity of the isolated phage to Tocilizumab. About 1.5 × 10^9^ pfu or 1.5 × 10^7^ pfu of the isolated phages were used.

### Phage competitive binding assay

Three sets of independent experiments were performed.

In two sets, ELISA plates were coated with Tocilizumab (0.5 μg/ml in 0.1 M NaHCO_3_, pH 8.6) overnight at 4°C. The plates were then washed with TBST and blocked by incubation with TBS containing 5 % dry milk. About 1.5 × 10^9^ pfu of purified phages in TBS containing 5 % dry milk were input and incubated for 1 h. After washing, the purified phages were eluted with 0.2 M Glycine–HCl (pH 2.2) and recombinant IL-6R (rIL-6R) (1 μg/ml, R&D Systems, Minneapolis, MN). An irrelevant peptide was used as an additional control. Then we performed the titration. The purified phages were also used to incubate with rIL-6R (1 μg/ml) for 1 h. Then the bound phage particles were detected with the peroxidase-conjugated mouse anti-phage M13 monoclonal antibody. The reaction was then developed with ABTS as a substrate. Absorbance was read at a wavelength of 405 nm with an ELISA reader.

In Set three, ELISA plates were coated with rIL-6R (0.1 μg/ml in 0.1 M NaHCO_3_, pH 8.6) overnight at 4°C. After the plates were washed and blocked, about 1.5 × 10^11^ to 1.5 × 10^7^ pfu of purified phage and Tocilizumab were co-incubated for 1 h. Then Tocilizumab was detected with Horseradish peroxidase (HRP)–conjugated Fc-specific goat ant-human IgG (Abcam, Cambridge, UK). The reaction was then developed with ABTS as a substrate. Absorbance was read at a wavelength of 405 nm with an ELISA reader. All assays were performed in triplicates.

### Synthesis of vaccine constructs

The peptide 4A124 (YHTTDKLFYMMRGGGS), peptide 4A125 (YSAYEFEYILSSGGGS), peptide 4A126 (KTMSAEEFDNWLGGGS), peptide 4A220 (LTSHTYRSQADTGGGS) and control peptide (MHSSFISPSALGGGS) were chemically synthesized (SBS, Beijing, China). Each peptide was then coupled through its C terminus to an immunogenic carrier, keyhole limpet hemocyanin (KLH, SIGMA, St. Louis, MO).

### Peptide competitive binding assay

ELISA plates were coated with rIL-6R (0.1 μg/ml in 0.1 M NaHCO_3_, pH 8.6) overnight at 4°C. After the plates were washed and blocked, synthesized peptides (10 μg/ml) and Tocilizumab (1 μg/ml) were co-incubated for 1 h. Then Tocilizumab was detected with HRP–conjugated Fc-specific goat ant-human IgG (Abcam, Cambridge, UK). The reaction was then developed with ABTS as a substrate. Absorbance was read at a wavelength of 405 nm with an ELISA reader.

### Immunization of BALB/c mice

Six groups (n = 8) of BALB/c mice were immunized by intraperitoneal injection with 100 μg of the 4A124 peptide-KLH, the 4A125 peptide-KLH, the 4A126 peptide-KLH, the 4A220 peptide-KLH, the control peptide-KLH, or KLH alone, at days 1, 22, 43 and 65. The complete Freund adjuvant/incomplete Freund adjuvant (CFA/IFA) (SIGMA, St. Louis, MO) was used as an adjuvant in all groups. Sera were obtained from the tail veins at day 0, 8, 29, 50 and 79. Ethical approval for use of animals in research was obtained from the Ethics Committee of Baotou Medical College. The animals were maintained in a pathogen-free facility in the Department of Laboratory Animal Science of Peking University Health Science Center. All procedures involving animals were performed according to the Research Animal Administration Guidelines of China and the Guidelines for the Care and Use of Laboratory Animals in China.

### Titer determination and Western blot assay

Serum antibody titers from the immunized BALB/C mice against rIL-6R were determined using general ELISA method. ELISA plates were coated with rIL-6R (10 μg/ml) overnight at 4°C. After the plates were washed and blocked, pooled serum were added and incubated for 2 h. Then bound antibodies were then incubated with HRP–conjugated Fc-specific goat ant-mouse IgG (Abcam, Cambridge, UK). The reaction was then developed with ABTS as a substrate. Absorbance was read at a wavelength of 405 nm with an ELISA reader. For antibody binding assays, aliquots of HeLa, U-937, Jurkat and FLS-RA cell lysates containing 50 μg protein were separated using sodium dodecyl sulfate-polyacrylamide gel electrophoresis and then electrophoretically transferred to polyacrylamide fluoride (PVDF) membranes. The membranes were blocked with PBS containing 5 % dry milk for 2 h at 37°C and then incubated with Tocilizumab (10 μg/ml) or pooled serum with a dilution 1:1,000 for overnight at 4°C. Bound antibodies were then incubated with IRDye 800CW-conjugated goat anti-human and anti-mouse antibody (LiCOR, Lincoln, NE) diluted with 1:10,000 for 1 h at room temperature. The membranes were scanned by the infrared imaging system (LiCOR, Lincoln, NE). For antibody competitive assays, aliquots of HeLa cell lysates containing 40 μg protein were separated using sodium dodecyl sulfate-polyacrylamide gel electrophoresis and then transferred to PVDF membranes. The membranes were blocked with PBS containing 5 % dry milk for 2 h at 37°C and then incubated with pooled serum with or without Tocilizumab overnight at 4°C. Bound antibodies were then incubated with IRDye 800CW-conjugated anti-mouse antibody. For signaling pathway detection, aliquots of cell lysates of treated HeLa, U937 and Jurkat cells containing 20 μg protein were separated using sodium dodecyl sulfate-polyacrylamide gel electrophoresis and then transferred to PVDF membranes. The membranes were blocked with PBS containing 5 % BSA for 2 h at 37°C and then reacted with antibodies against phosphorylated (Y705)-signal transducers and activator of transcription (STAT)3, phosphorylated -extracellular signal-regulated kinase (Erk) 1/2, STAT and Erk1/2 (Cell Signaling Technology, Beverly, USA). Bound antibodies were then incubated with IRDye 800CW-conjugated anti-rabbit antibody.

### Fluorescent immunostaining

HeLa, U-937, Jurkat and FLS-RA cells were plated on confocal dishes (Nest Biotechnology, Hongkong, China). The cells were grown overnight at 37°C and 5 % CO_2_. After washing with PBS, the cells were fixed with 4 % paraformaldehyde in PBS for 30 min. The confocal dishes were then blocked with 10 % goat serum in PBS for 1 h, followed by incubation with Tocilizumab (100 μg/ml), or the samples from the fourth immune sera with a dilution 1:200 and incubated at room temperature for 1 h. The bound antibodies were detected using fluorescein isothiocyanate (FITC)-conjugated goat anti-human IgG, and FITC-conjugated goat anti-mouse IgG (Abcam, Cambridge, UK) with a dilution 1:100 and DAPI (Roche, Shanghai, China) with a dilution 1:1000. The cells were observed with a fluorescence microscope (Olympus, Shanghai, China).

### Antibody-dependent cellular cytotoxicity (ADCC)

Hela, U937 and Jurkat cells were used as specific target cells. The pooled immune sera of the mimotope-immunized mice or the control mice from day 65 were diluted 1:100 for ADCC assays. Lymphocytes of the naive BALB/c mice (prepared by mashing the spleen and thymus) were prepared as effector cells. The effector to target (E: T) cell ratio was about 100:1. The target cells were incubated in a 96-well plate overnight, and then the pooled immune sera were added to the wells with the effector cells. After co-incubation for 72 h, the supernatants were removed carefully. The reaction was developed with the Cell Counting Kit-8 (Dojindo, Shanghai, China) diluted 1:10. The optical density at 490 nm was measured using an ELISA reader (Thermo Fisher Scientific, Shanghai, China).

### Statistical analysis

Data analyses were performed using GraphPad Prism 5.0 software. Results are expressed as the mean ± SD. Statistical differences were analyzed using the one-way ANOVA. *P* values less than 0.05 were considered significant.

## Results

### Biopanning and mimotopes characterization

**Fig. 1:**
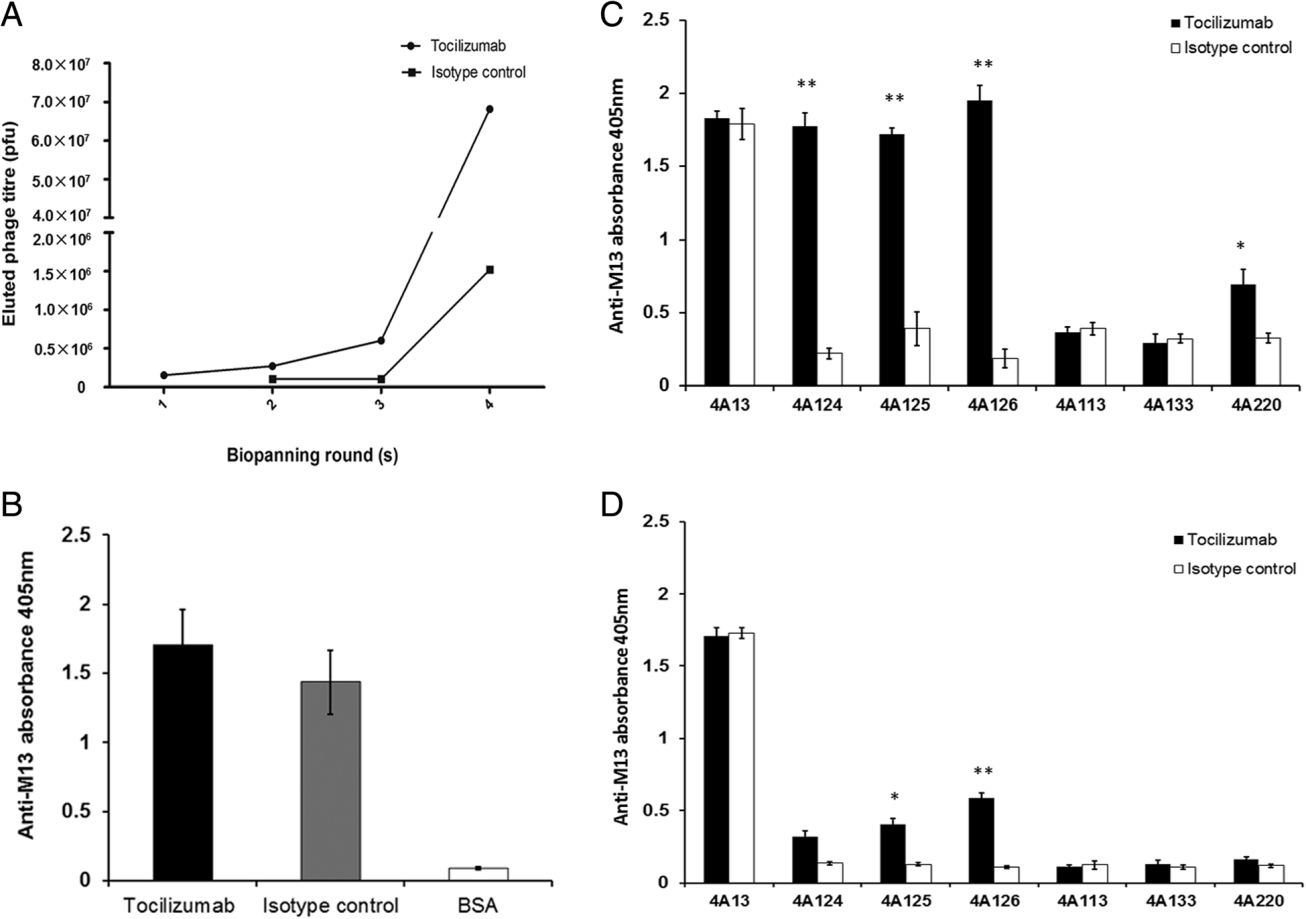
Four phage mimics were isolated and specifically recognized by Tocilizumab. Four rounds of biopanning were performed to identify the phage mimics. **a** The phage titer increased from 1.5 × 10^5^ pfu/ml (first round) to 2.7 × 10^5^ pfu/ml (second round) to 6.0 × 10^5^ (third round) finally to 6.8 × 10^7^ pfu/ml (fourth round). **b** Specificity ELISA was then performed to observe whether the whole fourth amplified phage could be specifically recognized by the Tocilizumab. The results showed the whole fourth amplified phage could be well recognized by both Tocilizumab and isotype control. Then 37 phage clones was selected and sequenced, and 7 different insert sequences were observed. Then we performed experiments to observe whether the seven different peptides displayed could be specifically recognized by the Tocilizumab. The results showed that four mimotope candidates were specifically recognized by Tocilizumab but not by the isotype-matched control antibody when 10^9^ pfu (**c**) or 10^7^ pfu (**d**) of the isolated phage particles were used (mean ± SD, *, P < 0.05; **, P < 0.005)

As Tocilizumab is a humanized monoclonal antibody that recognizes sIL-6R and mIL-6R, it was used as the target protein to screen peptides that can mimic the epitope. The eluted phage titrations increased with each round of panning, indicating that the phage particles might carry epitope mimics. The phage titrations were 1.5 × 10^5^ pfu/ml (first round), 2.7 × 10^5^ pfu/ml (second round), 6.0 × 10^5^ (third round) and 6.8 × 10^7^ pfu/ml (fourth round), respectively, suggesting the phage bounding to Tocilizumab was well enriched (Fig. 1A).

ELISA was then performed to decide whether the amplified phages could be specifically recognized by Tocilizumab. The results showed the amplified phages from 4 rounds of screening could be well recognized by both Tocilizumab and isotype controls, but no significant difference was observed (Fig. 1B).

Genomic DNAs from 37 phage clones were selected and sequenced, and seven different insert sequences were obtained. Then we performed experiments to observe whether the seven different peptides displayed could be specifically recognized by Tocilizumab. When about 1.5 × 10^9^ pfu of the isolated phages were used, the four mimotope candidates were specifically recognized by Tocilizumab but not by the isotype-matched controls (Fig. 1C). To decide which candidate exhibited better specificity, we reduced the input from 1.5 × 10^9^ to 1.5 × 10^7^pfu. Our results demonstrated that the phage clones 4A125 and 4A126 showed a better specificity, as compared to the other two (Fig. 1D).

**Fig. 2:**
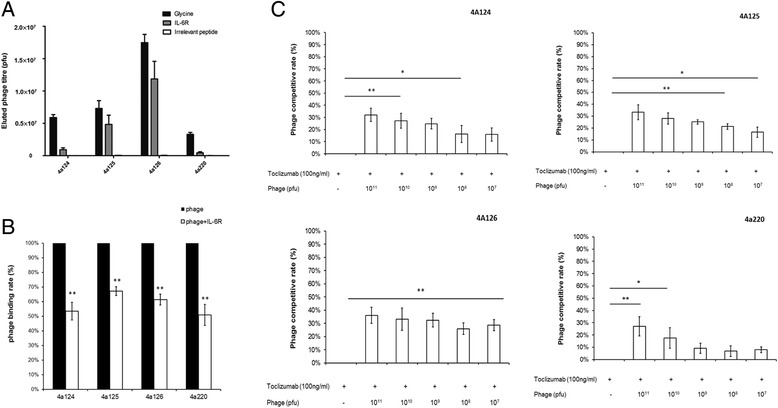
Isolated phage clones can mimic the original antigen. To demonstrate mimicry between the peptides and the original antigen, three independently phage competitive binding assay were then performed. **a** In Set one, rIL-6R could displace a large amount of bound phage from the Tocilizumab by using titration. **b** In Set two, the phage binding rate of all phage mimics decreased significantly after we added rIL-6R to compete with the binding of phage particles to Tocilizumab by using ELISA. **c** In Set three, the phage competitive rate of all phage mimics increased significantly after we added phage particles to compete with the binding of Tocilizumab to rIL-6R using ELISA (mean ± SD, *, P < 0.05; **, P < 0.005)

To demonstrate mimicry between the peptides and the original antigen, three independent phage competitive binding assay were then performed. In set one, 0.2 M Glycine–HCl (pH 2.2), rIL-6R and an irrelevant peptide were used to compete with the binding of phage particles to Tocilizumab. The bound phage particles were detected by titer determination. Recombinant IL-6R could displace a large amount of bound phage from the Tocilizumab, *i.e.*, the phage mimic 4A124 bound to Tocilizumab were displaced, about 5.9 × 10^6^ pfu using 0.2 M Glycine–HCl (pH 2.2), 9.3 × 10^5^ pfu using rIL-6R, only 1.0 × 10^4^ pfu using the irrelevant peptide, respectively. The phage mimic 4A125 bound to Tocilizumab were displaced, about 7.3 × 10^6^ pfu using 0.2 M Glycine–HCl (pH 2.2), 4.8 × 10^6^ pfu using rIL-6R, only 4.0 × 10^4^ pfu using the irrelevant peptide, respectively. The phage mimic 4A126 bound to Tocilizumab were displaced, about 1.7 × 10^7^ pfu using 0.2 M Glycine–HCl (pH 2.2), 1.2 × 10^7^ pfu using rIL-6R, only 4.0 × 10^4^ using the irrelevant peptide, respectively. The phage mimic 4A220 bound to Tocilizumab were displaced, about 3.3 × 10^6^ using 0.2 M Glycine–HCl (pH 2.2), 4.5 × 10^5^ using rIL-6R, only 1.6 × 10^4^ using the irrelevant peptide, respectively (Fig. 2A).

In set two, the phage binding rate of all phage mimics decreased significantly after we added rIL-6R to compete with the binding of phage particles to Tocilizumab. The binding rate decreased to 53.5 % (4A124), 67.1 % (4A125), 61.3 % (4A126) and 50.8 % (4A220), respectively. The percentage of binding rate was calculated using the following formula:

$$ \mathrm{phage}\;\mathrm{binding}\;\mathrm{rate}\%=\frac{Api- Ab}{Ap- Ab}\times 100\%, $$

where Ab is the background absorbance (both phage particles and rIL-6R were not added), Ap is the phage absorbance (phage was added) and Api is the absorbance of the competitive absorbance (both phage particles and rIL-6R were added). The assay was performed in duplicate (Fig. 2B).

In set three, the phage competitive rate of all phage mimics increased significantly after we added phage particles to compete with the binding of Tocilizumab to rIL-6R. The phage competitive rate of phage mimic 4A124 is 32.10 % (10^11^), 27.2 % (10^10^), 24.8 % (10^9^), 16.3 % (10^8^), 15. 9 % (10^7^), respectively. The phage competitive rate of phage mimic 4A125 is 33.30 % (10^11^), 28.10 % (10^10^), 25.21 % (10^9^), 21.4 % (10^8^), 15.62 % (10^7^), respectively. The phage competitive rate of phage mimic 4A126 is 36.10 % (10^11^), 33.30 % (10^10^), 32.50 % (10^9^), 26.00 % (10^8^), 28.70 % (10^7^), respectively. The phage competitive rate of phage mimic 4A220 is 28.10 % (10^11^), 18.40 % (10^10^), 10.10 % (10^9^), 6.8 % (10^8^), 7.9 % (10^7^), respectively. The percentage of competitive rate was calculated using the following formula:

$$ \mathrm{phage}\kern0.5em \mathrm{conpetitive}\kern0.5em \mathrm{rate}\%=\frac{Atp- Ab}{At- Ab}\times 100\%, $$

where Ab is the background absorbance (both phage particles and Tocilizumab were not added), At is the Tocilizumab absorbance (Tocilizumab was added) and Atp is the absorbance of the competitive absorbance (both phage particles and Tocilizumab were added) (Fig. 2C). The assay was performed in duplicate.

**Fig. 3:**
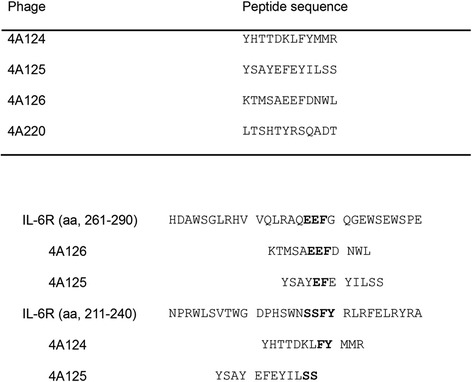
Alignment of the insert sequences expressed by phage clones. Phe229, Tyr230, Glu278 and Phe279 from the D3 domain of site I contribute the majority to form the initial IL-6/IL-6Rα binary complex. We aligned the mimotope sequences and found that phage mimics 4A124, 4A125 and 4A126 bear these key amino acids (4A124: Phe229, Tyr230; 4A125: Glu278, Phe279; 4A126: Glu278, Phe279)

Alignment of the insert sequences expressed by phage clones were shown in Fig. 3.

**Fig. 4:**
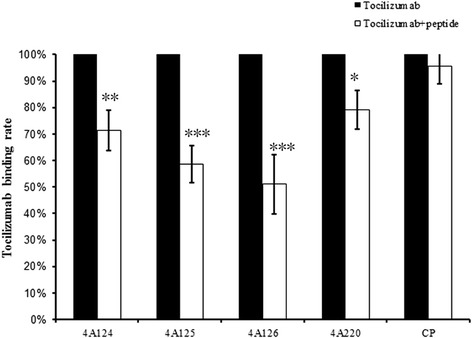
Peptides compete with rIL-6R to Tocilizumab. To demonstrate the peptides and original antigen can share the same binding site of Tocilizumab, peptide competitive binding assay was then performed. ELISA plates were coated with rIL-6R (0.1 μg/ml). After the plates were washed and blocked, synthesized peptides (10 μg/ml) and Tocilizumab (1 μg/ml) were co-incubated. Then Tocilizumab was detected with HRP–conjugated Fc-specific goat ant-human IgG. The results showed that Tocilizumab binding rate decreased significantly after we added peptides to compete with rIL-6R to Tocilizumab (mean ± SD, *, P < 0.05; **, P < 0.005; ***, p < 0.001)

Peptide competitive assay showed that the binding rate of Tocilizumab decreased significantly when we added peptides to compete the binding of Tocilizumab to rIL-6R. The binding rate decreased to 71.40 % (4A124), 58.60 % (4A125), 51.10 % (4A126), 9.10 % (4A220) and 95.50 % (CP), respectively. The percentage of binding rate was calculated using the following formula:

$$ \mathrm{phage}\kern0.5em \mathrm{binding}\kern0.5em \mathrm{rate}\%=\frac{Apt- Ab}{At- Ab}\times 100\%, $$

where Ab is the background absorbance (both peptides and Tocilizumab were not added), At is the Tocilizumab absorbance (Tocilizumab was added) and Apt is the absorbance of the competitive absorbance (both peptides and Tocilizumab were added). The assay was performed in duplicate (Fig. 4).

### Immune responses induced by mimotopes vaccination

**Fig. 5:**
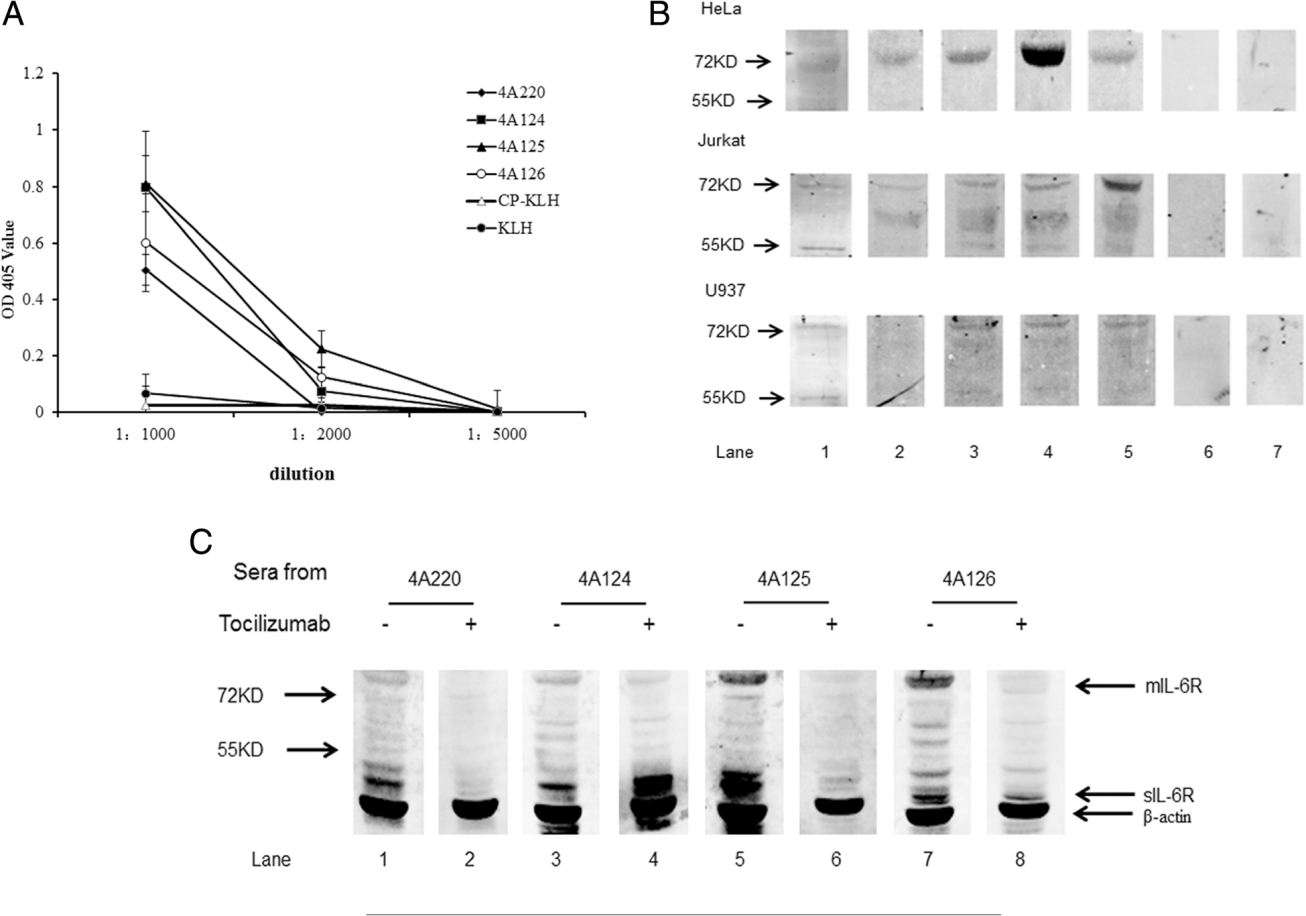
Mimotope-induced antibodies for IL-6R recognition. **a** Titers of the six mouse groups determined by ELISA (the fourth immune serum, day 79) against rIL-6R. **b** HeLa, Jurkat and U-937 cell lysates were loaded with 50 μg/per lane. IL-6R expressed in HeLa, Jurkat and U-937 detection using Tocilizumab (lane 1) and the sera from mice immunized with the 4A220-KLH conjugate (lane 2), the 4A124-KLH conjugate (lane 3), 4A125-KLH conjugate (lane 4), 4A126-KLH conjugate (lane 5), the control peptide-KLH conjugate (lane 6), or the carrier KLH alone (lane 7). Sera were diluted with 1:1000. The results showed that the antibodies in the sera of the mice immunized with mimotopes could recognize sIL-6R (55KDA) and/or mIL-6R (80KDA) expressed in HeLa, Jurkat and U-937 cell line. **c** For antibody competitive assays, HeLa cell lysates were loaded with 40 μg/per lane. IL-6R expressed in HeLa was detected by using sera from mice immunized with the 4A220-KLH conjugate (lane 1), Tocilizumab+ sera from mice immunized with the 4A220-KLH conjugate (lane 2), sera from mice immunized with the 4A124-KLH conjugate (lane 3), Tocilizumab+ sera from mice immunized with the 4A124-KLH conjugate (lane 2), sera from mice immunized with the 4A125-KLH conjugate (lane 5), Tocilizumab+ sera from mice immunized with the 4A125-KLH conjugate (lane 2), sera from mice immunized with the 4A126-KLH conjugate (lane 7) and Tocilizumab+ sera from mice immunized with the 4A220-KLH conjugate (lane 8). Sera were diluted with 1:1000. The results showed that antibodies induced by mimotopes-KLH conjugate could compete with Tocilizumab to IL-6R expressed in HeLa cells, except antibodies induced by 4A124-KLH conjugate

**Fig. 6:**
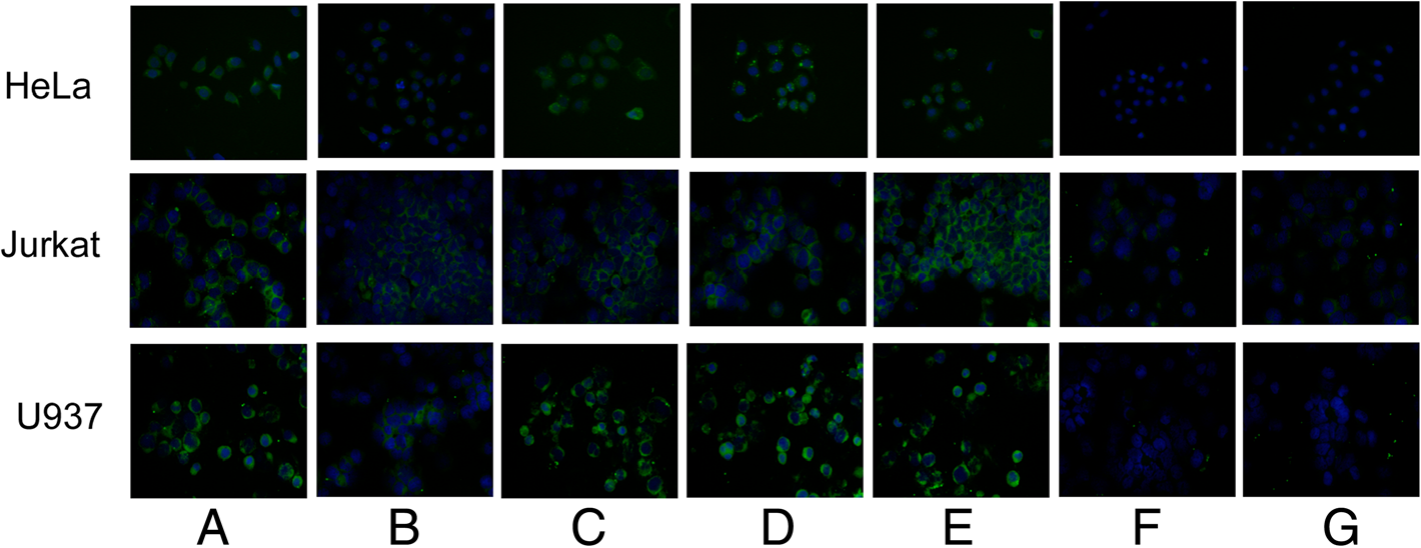
Immunofluorescence staining of IL-6R expressed in Hela, U-937 and Jurkat cell line. Cells were incubated with Tocilizumab and pooled serum followed by FITC-labeled secondary antibodies where the nuclei were stained with DAPI. Cells were visualized with a fluorescence microscope. **a** Tocilizumab, (**b**) sera from mice immunized with 4A220-KLH, (**c**) sera from mice immunized with 4A124-KLH, (**d**) sera from mice immunized with 4A125-KLH, (**e**) sera from mice immunized with 4A126-KLH, (**f**) sera from mice immunized with the control peptide-KLH, (**g**) sera from mice immunized with KLH alone

From the specificity ELISA and the competitive binding assay results, the four reactive peptides (4A124, 4A125, 4A126 and 4A220) displayed by phage particles were chosen for chemical synthesis. The immunogenicity of the mimotope conjugates was then evaluated by immunizing BALB/c mice. Mice immunized with the mimotope conjugates successfully developed antibodies against rIL-6R (Fig. 5A) and sIL-6R (55 kDa) and/or mIL-6R (80 kDa) expressed in Hela, U-937 and Jurkat cells (Fig. 5B). Antibody competitive assays showed that Tocilizumab could compete with antibodies induced by 4A220, 4A125 and 4A126-KLH conjugate for IL-6R. However, we noticed that more antibodies induced by 4A124-KLH conjugate bound to sIL-6R when incubated with Tocilizumab (Fig. 5C). Results of fluorescent immunostaining revealed that mimotopes induced mouse antibodies could recognize IL-6R in Hela, U-937 and Jurkat cell line, as compared to the control groups (Fig. 6).

### Signaling pathway analysis

**Fig. 7:**
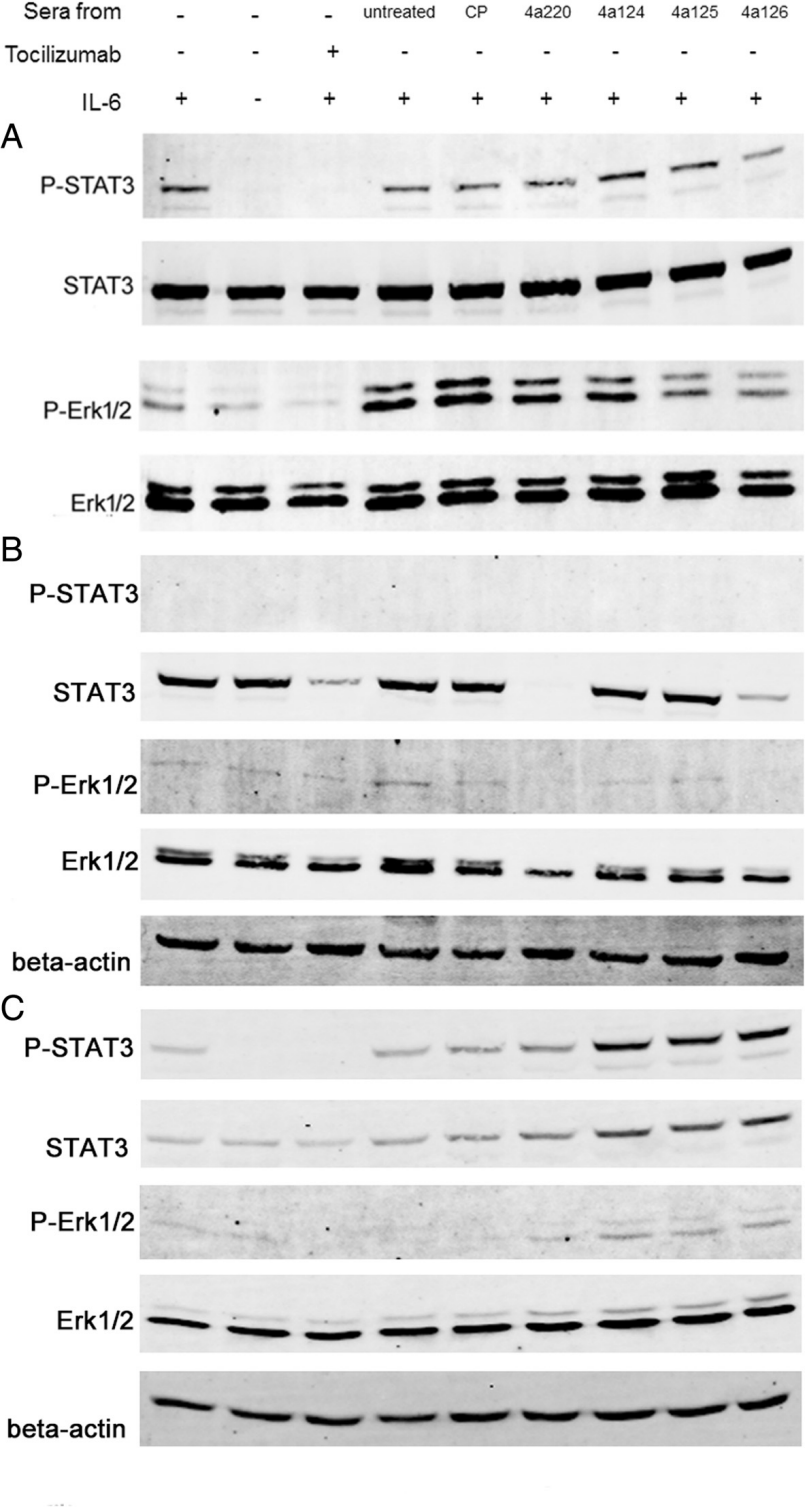
Signaling pathway analysis with mimotope-induced antibodies. We detected the levels of phosphorylated-STAT3, STAT3, phosphorylated-Erk1/2 and Erk1/2 in HeLa, Jurkat and U937 cells to observe whether IL-6 pathway could be down-regulated by when added sera from mice immunized with mimotopes. Cells were grown at 37 °C with medium containing 10 % FBS. Two days later, the medium was replaced by FBS-free medium with sera from mimotopes immunized mice with a dilution 1:100 or Tocilizumab (10 μg/ml) at 37 °C for 2 h. Then the cells were incubated with human IL-6 (40 ng/ml) for 15 min at 37 °C. In HeLa cells (**a**), incubation with sera from mice immunized with 4A125 and 4A126 could partly reduce the levels of phosphorylated-STAT3 and phosphorylated-Erk1/2, as compared to the control group. In Jurkat cells (**b**), incubation with sera from mice immunized with 4A220 and 4A126-KLH conjugate could reduce the level of STAT3, phosphorylated-Erk1/2 and Erk1/2. In U937 cells (**c**), incubation with sera from mice immunized with 4A124, 4a125 and 4A126-KLH conjugate could increase the level of phosphorylated- STAT3, STAT3 and phosphorylated-Erk1/2

We next addressed the question whether IL-6 pathway could be down-regulated by adding sera from mice immunized with Tocilizumab mimotopes. In HeLa cells, incubation with sera from mice immunized with 4A125 and 4A126-KLH conjugate could reduce the level of phosphorylated- STAT3 and phosphorylated-Erk1/2, as compared to the control group. In Jurkat cells, incubation with sera from mice immunized with 4A220 and 4A126-KLH conjugate could reduce the level of STAT3, phosphorylated-Erk1/2 and Erk1/2. In U937 cells, incubation with sera from mice immunized with 4A124, 4a125 and 4A126-KLH conjugate could increase the level of phosphorylated- STAT3, STAT3 and phosphorylated-Erk1/2 (Fig. 7).

### ADCC induced by antibodies against mimotopes

To assess whether the antibodies induced by the mimotopes can exhibit specific lysis of the target cells, we performed ADCC assay. The percentage of cellular cytotoxicity was calculated using the following formula:

$$ \%\mathrm{cytotoxicity}=1-\left[\frac{Ae- Ab}{Au- Ab}\right]\times 100\%. $$

**Fig. 8:**
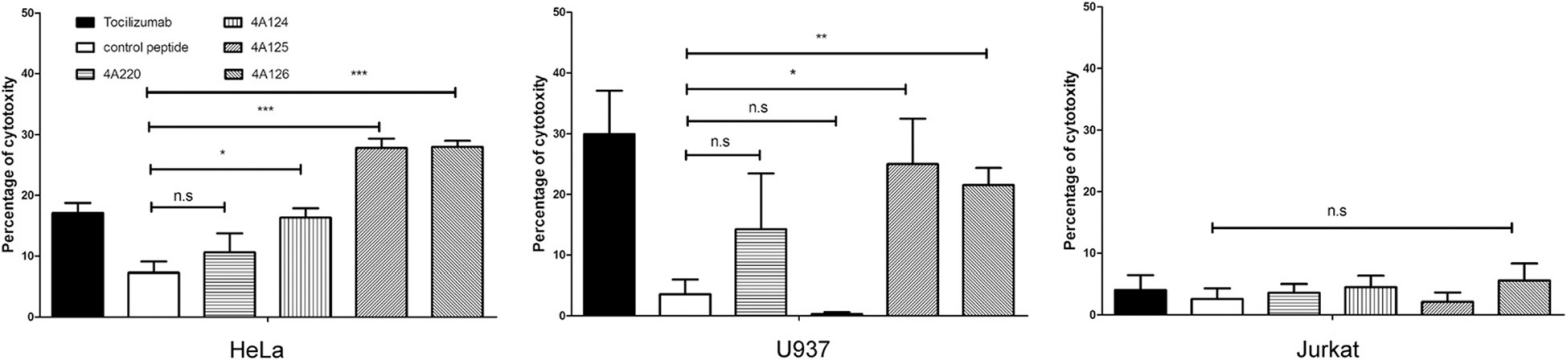
ADCC against HeLa, U937 and Jurkat cells elicited by mimotope-induced antibodies. To assess whether the antibodies induced by the mimotope conjugates can exhibit specific lysis of the target cells, we performed the ADCC assay. The fourth pooled immune sera of the mimotope-immunized mice or the control mice were diluted 1:100 and used in ADCC assays. Lymphocytes of the naive BALB/c mice were prepared as effector cells. The effector to target (E: T) cell ratio was about 100:1. The results showed that the antibodies induced by 4A124, 4A125 and 4A126-KLH conjugate could elicite specific lysis of HeLa and U937 cells. However, no specific lysis was observed in Jurkat cells (mean ± SD, *, P < 0.05; **, P < 0.005; ***, p < 0.001)

The results showed that the antibodies induced by 4A220, 4A124, 4A125 and 4A126-KLH conjugate elicited specific lysis of 10.59 %, 16.33 %, 27.80 % and 27.98 % of HeLa cells, while the antibodies induced by 4A220, 4A124, 4A125 and 4A126-KLH conjugate elicited specific lysis of 14.23 %, 0.31 %, 25.01 % and 21.55 % of U937 cells. In Jurkat cells, the antibodies induced by 4A220, 4A124, 4A125 and 4A126 elicited specific lysis of 3.59 %, 4.51 %, 2.12 % and 5.59 % (Fig. 8).

### Antibodies induced by mimotopes binding to IL-6R of FLS-RA

**Fig. 9:**
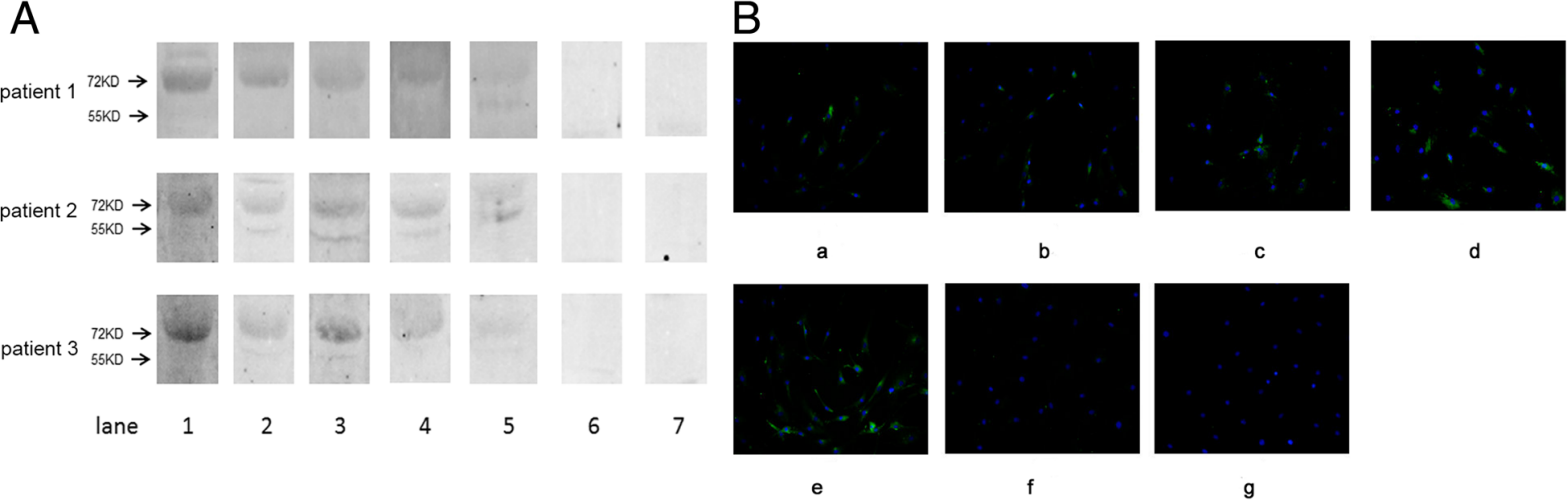
Western blot and immunofluorescence staining with mimotope-induced antibodies for IL-6R recognition in FLS-RA. **a** Fibroblast-like synoviocytes lysates from three patients with RA were loaded with 40 μg/per lane. IL-6R expressed in FLS-RA detection using Tocilizumab (lane 1) and the sera from mice immunized with the 4A220-KLH conjugate (lane 2), the 4A124-KLH conjugate (lane 3), 4A125-KLH conjugate (lane 4), 4A126-KLH conjugate (lane 5), the control peptide-KLH conjugate (lane 6), or the carrier KLH alone (lane 7). Sera were diluted with 1:1000. **b** Represent image of IL-6R recognition in FLS-RA. FLS-RA cells were incubated with Tocilizumab and pooled serum followed by FITC-labeled secondary antibodies where the nuclei were stained with DAPI. (a) sera from mice immunized with 4A220-KLH, (b) sera from mice immunized with 4A124-KLH, (c) sera from mice immunized with 4A125-KLH, (d) sera from mice immunized with 4A126-KLH, (e) Tocilizumab, (f) sera from mice immunized with the control peptide-KLH, (g) sera from mice immunized with KLH alone. The results showed that the antibodies in the sera of the mice immunized with mimotopes could recognize IL-6R expressed in FLS-RA

Then we detected whether the antibodies induced by mimotopes could bind to the FLS-RA cell lysates. The results of western bolt (Fig. 9A) and fluorescent immunostaining (Fig. 9B) showed that the antibodies in the sera of the mice immunized with mimotopes could recognize sIL-6R (55 kDa) and/or mIL-6R (80 kDa) expressed in FLS-RA.

## Discussion

One way to overcome the serious practical limitations of the monoclonal antibody approach could be to perform immunization with peptide mimics (mimotopes) of a naturally occurring epitope that is recognized by the desired antibody. To isolate mimotopes of Tocilizumab, which has been considered as a promising agent for treating RA, the phage display was exploited in this study. After four rounds of biopanning, we isolated four peptide mimics that were specifically recognized by Tocilizumab. IL-6 is the prototypic gp130-cytokine and binds the gp130 receptor through three conserved epitopes (sites I, II, and III), of which site III is unique to gp130-cytokines. IL-6 must first form a complex with a nonsignaling α receptor, IL-6Rα (PDB: 1N26_A), through site I. Phe229, Tyr230, Glu278 and Phe279 from the D3 domain of site I contribute the majority to form the initial IL-6/IL-6Rα binary complex [20, 21]. We aligned the mimotope sequences and found that phage mimics 4A124, 4A125 and 4A126 bear these key amino acids (4A124: Phe229, Tyr230; 4A125: Glu278, Phe279; 4A126: Glu278, Phe279), suggesting that not only Tocilizumab but also the antibodies induced by these mimotopes may prevent IL-6 binding to IL-6Rα through these key amino acids.

Titer determination was performed to evaluate the immunogenicity of the isolated mimotopes. As small peptide molecules are not sufficient enough to stimulate the immune system and may even induce tolerance, we coupled the mimotopes to an immunogenic carrier, KLH. Mice immunized with 4A124, 4A125, 4A126 and 4A220 successfully developed high antibody titers against KLH as well as the mimotopes. The induced sera also recognized rIL-6R and IL-6R expressed in Hela, U-937 and Jurkat cell line. Western blots and immunofluorescence staining further supported that the antibodies induced by the four isolated mimotopes can bind to IL-6R. Notably, we found that more antibodies induced by 4A124 bound to sIL-6R when incubated with Tocilizumab. We assumed that the antibodies induced by 4A124 may bind both sIL-6R and Tocilizumab, so Tocilizumab could act like “bridge” to help antibodies induced by 4A124 to bind sIL-6R.

In signaling pathway analysis, we found that antibodies induced by mimotopes play different roles in different cell line. In HeLa and Jurkat cells, antibodies induced by mimotopes may act as a neutralizing role, but in U937 cells, they may have an aggressive role. Notably, when we added IL-6 with sera from untreated or CP mice, the level of phosphorylated-Erk1/2 increased more than IL-6 alone in HeLa cells. Why the level of phosphorylated-Erk1/2 phosphorylation increased more when adding IL-6 with sera? We assumed that the most likely explanation was that some homologous cytokines might overlap human IL-6 to activate signaling pathway in HeLa cells. In addition, we found that antibodies induced by 4A220 and 4A126-KLH conjugate and Tocilizumab could reduce the level of total STAT3, phosphorylated-Erk1/2 and total Erk1/2 in Jurkat cells. The mechanism how anti-IL-6R antibodies could reduce the level of total STAT3 and Erk1/2 in Jurkat cells needs further study.

In ADCC assays, we found that anti-sera of all mimotopes could not induce ADCC effect against Jurkat cells. Because Jurkat cells are apt to form colony, we assumed that the effector cells could not contact to inner target cells and show significantly specific lysis to Jurkat cells.

The antibodies induced by all the phage mimics show a similar capacity of binding the IL-6R expressed in Hela, U-937, Jurkat and FLS-RA cells. We noticed that mimotope 4A220 may not bear any key amino acids, but the antibodies induced by 4A220 still recognized IL-6R in these cell lines. We assumed that although mimotope 4A220 is composed of different amino acids, it may structurally mimic the Tocilizumab-binding site.

Our study demonstrated that active immunization induced by Tocilizumab mimotopes *in vivo* can produce the desired antibody. With active immunization, the main obstacles of passive immunotherapy, specifically the comparatively short antibody half-life, may be overcome. Immunological memory will be induced to provide ongoing protection for vaccinated individuals. Meanwhile, the immunogenicity of artificial antibodies may be solved because the antibodies are produced by the patients themselves. To further evaluate the feasibility of mimotopes vaccination approach, the mimotopes of AM16-1 (mAb against murine IL-6R) should be screened and used to immunize the collagen-induced arthritis (CIA) animal models for us to observe whether the mimotopes of AM16-1 could raise antibodies against the murine IL-6R and ameliorate the arthritis in animal models.

## Conclusions

We successfully isolated four mimotopes of Tocilizumab and demonstrated that active immunization induced by these mimotopes can produce the desired antibody *in vivo*. Mimotopes identified for IL-6R may represent a novel alternative for treating RA or other autoimmune diseases.

## Abbreviations

IL-6: Interleukin-6
sIL-6R: Soluble interleukin-6 receptor
mIL-6R: Membrane bound interleukin-6
mAb: Monoclonal antibody
ELISA: Enzyme-linked immunosorbent assay
FLS-RA: Fibroblast-like synoviocytes from patients with RA
RA: Rheumatoid arthritis
sJIA: Systemic juvenile idiopathic arthritis
RANKL: Receptor activator of nuclear factor-Κb ligand
VEGF: Vascular endothelial growth factor
HRP: Horseradish peroxidase
ABTS: 2, 2-azino-bis-(3-ethylbenzthiazoline-6-sulphonic acid
KLH: Keyhole limpet hemocyanin
CFA/IFA: Complete Freund adjuvant/incomplete Freund adjuvant
CP: Control peptide
PVDF: Polyacrylamide fluoride
FBS: Fetal bovine serum
BSA: Bovine serum albumin
CIA: Collagen-induced arthritis
STAT3: Signal transducers and activator of transcription
Erk: Phosphorylated- extracellular signal-regulated kinase
